# Further evidence against the role renal medullary perfusion in short-term control of arterial pressure in normotensive and mildly or overtly hypertensive rats

**DOI:** 10.1007/s00424-021-02534-1

**Published:** 2021-03-02

**Authors:** Bożena Bądzyńska, Iwona Baranowska, Janusz Sadowski

**Affiliations:** Department of Renal and Body Fluid Physiology, Mossakowski Medical Research Institute, Polish Academy of Sciences, 5 Pawińskiego St., 02-106 Warsaw, Poland

**Keywords:** Arterial blood pressure, Arterial hypertension, DOCA-salt hypertension, Norepinephrine, Renal medullary circulation

## Abstract

Earlier evidence from studies of rat hypertension models undermines the widespread view that the rate of renal medullary blood flow (MBF) is critical in control of arterial pressure (MAP). Here, we examined the role of MBF in rats that were normotensive, with modest short-lasting pressure elevation, or with overt established hypertension. The groups studied were anaesthetised Sprague-Dawley rats: (1) normotensive, (2) with acute i.v. norepinephrine-induced MAP elevation, and (3) with hypertension induced by unilateral nephrectomy followed by administration of deoxycorticosterone-acetate (DOCA) and 1% NaCl drinking fluid for 3 weeks. MBF was measured (laser-Doppler probe) and selectively increased using 4-h renal medullary infusion of bradykinin. MAP, renal excretion parameters and post-experiment medullary tissue osmolality and sodium concentration were determined. In the three experimental groups, baseline MAP was 117, 151 and 171 mmHg, respectively. Intramedullary bradykinin increased MBF by 45%, 65% and 70%, respectively, but this was not associated with a change in MAP. In normotensive rats a significant decrease in medullary tissue sodium was seen. The intramedullary bradykinin specifically increased renal excretion of water, sodium and total solutes in norepinephrine-treated rats but not in the two other groups. As previously shown in models of rat hypertension, in the normotensive rats and those with acute mild pressure elevation (resembling labile borderline human hypertension), 4-h renal medullary hyperperfusion failed to decrease MAP. Nor did it decrease in DOCA-salt model mimicking low-renin human hypertension. Evidently, within the 4-h observation, medullary perfusion was not a critical determinant of MAP in normotensive and hypertensive rats.

## Introduction

Despite a substantial progress in the treatment of arterial hypertension over the past three decades, the effectiveness of the available therapeutic measures, including the novel approach based on catheter-based renal denervation [3, 30], is still limited and cannot be regarded as satisfactory. Multiple forms of human primary hypertension have been identified, with a variety of pathogenetic mechanisms underlying or contributing to the development of the final phenomenon, i.e. an increase in systemic vascular resistance. As reviewed recently [28], the pathogenesis of hypertension is still a matter of debate, mostly between the proponents of “renocentric” theory rooted in the seminal work of Guyton and his adherents [13, 22, 23] and the researchers who propose that the origin of the phenomena leading to hypertension is in the brain and the nervous system in general (“neurocentric” theory), with a special role ascribed to activity of the sympathetic nervous system, at its different levels [17, 21, 24, 27]. Over the past decade, the very basic principles of the renocentric concept have been ardently criticised [2, 5, 6, 27, 32]. Therefore, attempts are made to reconcile the opposed views and incorporate the well-documented elements of the neurocentric and renocentric theories to develop an integrative concept [8, 9, 18, 19].

Within the renocentric concept, the postulated crucial events in the mechanisms which correct elevation of BP include an increase in (allegedly poorly autoregulated) medullary blood flow (MBF) leading to increased renal interstitial hydrostatic pressure, first within the medulla and then throughout the kidney (including the proximal peritubular space), followed by a change in Starling forces and inhibition of tubular transport and the development of natriuresis. Considering the effect per se of increasing *vasa recta* blood flow, an additional mechanism for inhibition of tubular transport could be the phenomenon of wash-out of solutes from the medullary interstitium and reduction the medullary/papillary osmotic hypertonicity, leading to inhibition of fluid reabsorption along the medullary tubule segments [7, 14, 15, 25]. However, substantial evidence from animal experiments, as reviewed recently [28] and also a recent report from a patient study [1], contradict the causal role of MBF in the process.

In an earlier study with three variants of rat hypertension [11], we showed that experimental elevation of MBF had no rapid effect on BP, excluding thereby a possible role of medullary vasodilator (medullipin) allegedly released within less than 1 h after MBF elevation, as postulated by Muirhead [26]. More recently, we showed that also longer lasting (4 h) medullary hyperperfusion failed to decrease BP in three models of rat hypertension [10].

In the present work, we first tested the hypothesis that elevation of medullary perfusion using intramedullary infusion of bradykinin (Bk) would lower arterial pressure in normotensive Sprague-Dawley rats (group: S-D) within a time interval (4 h) that would not be compatible with natriuresis and extracellular volume contraction as a causal factor. Normotensive rats were not studied in our earlier work [10] so such investigation was needed, given that the rate of medullary perfusion has long been proposed to be involved in hour-to-hour maintenance of normal arterial pressure [13, 22]. We also thought it important to compare the results in normotensive rats with those in the rats of the same strain with acute modest elevation of blood pressure obtained with NE infusion (group S-D+NE). The purpose was to examine the effect of pressure elevation per se, hopefully without mobilizing secondary pathogenetic mechanisms which, in the long run, would actually result in creation of a complex model of arterial hypertension. We assumed this approach would provide a situation which resembles transient elevations of arterial pressure observed in patients with labile or borderline hypertension.

In the next step, we wished to examine the effect of medullary hyperperfusion in established hypertension, a DOCA-salt (deoxycorticosterone plus 1% NaCl drinking) model, a variant of salt-dependent hypertension yet one with pronounced neurogenic pathogenetic component. This model has not been explored in our earlier studies of the role of renal medullary perfusion in BP control. It is emphasized that, given the limited duration of experimental medullary hyperperfusion, the results of this study pertain to hour-to-hour control of arterial pressure. Obviously, such control is unrelated to the mechanism dependent on natriuresis sustained over days and followed by extracellular fluid volume contraction.

## Material and Methods

### Ethical approval

The experimental procedures were approved by the extramural Second Local Ethical Committee for Animal Experimentation, Warsaw.

Male Sprague-Dawley (S-D) rats were derived from the animal house of the Mossakowski Medical Research Institute, Polish Academy of Sciences. Experiments were designed to examine whether in normotensive or hypertensive rats a selective increase in renal medullary perfusion (MBF) induced by intramedullary infusion of bradykinin (Bk) would lower mean arterial blood pressure (MAP), medullary tissue osmolality, and sodium concentration, and if it affects renal excretion.

Acute experiments were performed with male anaesthetised S-D rats, aged 12 weeks, weighing 280–340 g, maintained on standard sodium diet (0.25% Na^+^ w/w, SSNIFF, GmbH, Soest, Germany), with free access to water. The groups studied were as follows: (1) normotensive S-D rats, Bk: *n* = 8, solvent: *n* = 6; (2) S-D rats given intravenous norepinephrine infusion to elevate baseline MAP to about 150 mmHg (S-D+NE, Bk: *n* = 7, solvent: *n* = 8); (3) S-D rats with hypertension induced by initial uninephrectomy followed by replacing drinking water with 0.9% NaCl and application of deoxycorticosterone acetate (DOCA, 50 mg pellet implanted subcutaneously, the contents released over 21 days, Bk: *n* = 7, solvent: *n* =7). Experiments were performed 21 days after the implantation (DOCA-salt model).

### Surgical preparations

All experiments were performed in uninephrectomized rats. In S-D normotensive rats and S-D+NE rats, right-side nephrectomy was performed at the start of the acute experiment. DOCA-salt rats were subjected to right-side nephrectomy under inhalation anaesthesia (4% isoflurane/oxygen mixture) 2 weeks before acute experiment. Early nephrectomy is needed to effectively increase blood pressure in this hypertension model. Metacam (0.4 mg kg^−1^ BW, Boehringer, Ingelheim, Germany) and baytril (10 mg kg^−1^ BW, Bayer, Leverkusen, Germany) were given subcutaneously for analgesia and to prevent infection, respectively. Nephrectomy was performed from a flank incision, and care was taken to leave the right adrenal gland intact.

In acute experiments, the rats were anaesthetised with thiopental (Sandoz GmbH, Kundl, Austria), 100 mg kg^−1^ i.p., which provided stable anaesthesia for at least 5 h, with additional small subcutanaeous doses when needed. The jugular vein was cannulated for infusion of fluids and drugs. A polyethylene tube was placed in the trachea to ensure free airways. Body temperature was maintained at about 37 °C by means of a servo-controlled heating pad. During surgery, to maintain plasma volume, fluid losses were compensated by intravenous infusion of 3% bovine albumin in Ringer’s solution at 2.5 ml/h. With surgery completed within about 30 min, this infusion was replaced by 0.9% NaCl, with addition of norepinephrine (NE) in the appropriate group, given at the same volume infusion rate of 2.5 ml/h. For measurement of mean arterial blood pressure (MAP), a Teflon catheter was introduced into the right femoral artery and connected with a Stoelting blood pressure meter (Stoelting, Wood Dale IL, USA).

The left kidney was exposed from a subcostal flank incision and immobilized in a lucite cup, and the ureter was cannulated for timed urine collection to measure urine flow (V), sodium excretion (U_Na_V), and total solute excretion (U_osm_V). To measure total renal blood flow (RBF), a Transonic cuff probe was placed on the renal artery and connected with a Transonic flowmeter (Type T106, Transonic System Inc. Ithaca, NY, USA). Blood perfusion of the renal medulla (MBF) was measured as laser-Doppler flux using Periflux 4001 system (Perimed, Jarfalla, Sweden). A 32-gauge stainless steel cannula, connected with an infusion pump, was inserted into the kidney, with the tip located at the outer-inner medullary border.

### Protocols

In each protocol, an intramedullary infusion of 0.9% NaCl bradykinin (Bk) solvent was started at a volume rate of 0.5 ml h^−1^, followed by Bk infusion in experimental periods.

In S-D+NE rats, norepinephrine was infused to the jugular vein (at 0.83 mg h^−1^ kg^−1^ BW, i.v.) starting immediately after surgery and continued until the end of the experiment. In a preliminary group (*n* = 6), the effect of NE on blood pressure, renal haemodynamics and renal excretory function was first determined, without later application of Bk.

With the surgery completed, 1 h was allowed for equilibration of the haemodynamic parameters and urine excretion, followed by a 30-min control measurement period and urine collection. Subsequently, intramedullary solvent infusion was replaced by Bk (Sigma-Aldrich, Basel, Switzerland) solution given at 0.27 mg h^−1^ kg^−1^ BW during 4 h. Ten minutes later, MAP, RBF and MBF recording was started and continued throughout the experiment, along with 30-min urine collections.

In parallel time-control experiments, Bk solvent was infused throughout the experiment, with measurements performed as usual. At the end of all experiments, the left kidney was removed and the inner renal medulla was excised for measurement of tissue osmolality and sodium concentration [Na^+^]. The rats were killed with an overdose of thiopental.

### Determination of osmolality and sodium concentration ([Na^+^]) in renal inner medulla

After the experiment, the kidney was excised, sectioned along the sagittal paracentral plane in a way to preserve the inner (white) medulla intact. To obtain from the excised white medulla a standard medullary piece, the tissue was punched out using a stainless steel tube slightly oval at the cross section. The pieces included most of the white medulla but not the tip region of the papilla. They were weighed and subjected to 1-h extraction in a 1-ml volume of boiling distilled water and osmolality and sodium concentration of the extraction fluid (equilibrated with tissue fluid) was determined. The final results were expressed as mosmol and milimol Na per kg of wet medullary tissue [16].

### Analytical methods

Urine volume was determined gravimetrically. Urine and tissue osmolality were measured using a cryoscopic osmometer (Osmomat 030, Gonotec GmbH, Berlin, Germany). Urine and tissue sodium concentrations were determined using the flame photometer (Jenway PFP7, Essex, UK).

### Statistics

Data are expressed as means ± SEM. Significance of changes within one group over time was first evaluated by repeated measures analysis of variance (ANOVA), followed by modified Student *t* test for dependent variables, using Bonferroni correction for multiple comparisons [31]. Differences between profiles for bradykinin and solvent infusion were first analyzed by the repeated measurements multivariable ANOVA, followed by Duncan’s test (STATISTICA 10.0, StatSoft Polska Inc.). *P* ≤ 0.05 was taken to indicate significance of differences.

## Results

Baseline values of the mean arterial pressure (MAP) and renal haemodynamic and excretion parameters in normotensive S-D rats, in S-D + NE group and in DOCA-salt hypertensive rats are presented in Table 1. The data present the values for the left kidney since all the rats had undergone contralateral nephrectomy: the S-D and S-D+NE groups at the start of an acute experiment and the DOCA-salt rats 2 weeks earlier. Since in the latter group a substantial left kidney hypertrophy was seen, the renal blood flow, V, U_Na_V and U_osm_ V values are expressed per gram kidney weight (KW).

**Table 1:** Baseline values of mean arterial pressure, renal haemodynamics and renal excretion parameters and kidney weight (KW) in S-D normotensive rats, in norepinephrine-induced blood pressure elevation and in DOCA-salt hypertensive rats

	MAP	RBF	MBF	V	U_Na_V	U_osm_V	KW
*n*	mmHg	ml min^−1^ g^−1^KW	PU	μl min^−1^ g^−1^ KW	μmol min^−1^ g^−1^ KW	μosm min^−1^ g^−1^ KW	g
S-D (14)	117 ± 1	7.7 ± 0.5	137 ± 16	3.9 ± 0.4	0.4 ± 0.04	5.4 ± 0.4	1.41 ± 0.05
S-D+NE (15)	151 ± 2	6.1 ± 0.3	115 ± 10	22.6 ± 6.3	0.7 ± 0.2	8.6 ± 0.9	1.34 ± 0.03
DOCA-salt (14)	171 ± 4	4.5 ± 0.7	93 ± 10	13.8 ± 2.9	1.5 ± 0.3	6.8 ± 1.0	3.14 ± 0.08

MAP mean arterial pressure, RBF renal blood flow, MBF renal medullary perfusion, excretion of water (V), sodium (U_Na_V) and total solutes (U_osm_V). Pooled control data from Bk and solvent infusion studies, n values are given in parentheses. Groups: normotensive Sprague-Dawley (S-D) rats; norepinephrine-induced hypertension (S-D+NE); DOCA-salt hypertensive rats.

It is seen that, on the average, NE infusion induced an acute elevation of BP by 34 mmHg (+ 29%) whereas in DOCA-salt hypertension model, the pressure was 54 mmHg above the level for control S-D rats (+ 46%). By contrast, RBF and MBF showed an inverse (decreasing) trend pattern in the respective groups. Therefore, in SD+NE and DOCA-salt rats, the renal vascular resistance can be estimated to be, respectively, about 1.6-fold and 2.6-fold higher than in S-D normotensive rats.

### Normotensive rats

**Fig. 1:**
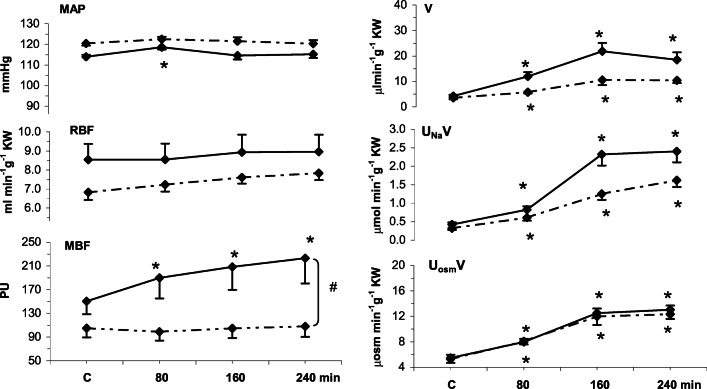
Responses of mean arterial blood pressure, renal haemodynamics and excretion parameters to 4-h renal intramedullary infusion of bradykinin (Bk) or solvent in normotensive rats. MAP, mean arterial pressure; RBF, renal blood flow; MBF, renal medullary perfusion; excretion of water (V), sodium (U_Na_V) and total solutes (U_osm_V). Means ± SEM: averaged 30-min values measured in the control period (C) and at the end of 80, 160 and 240 min of Bk infusion. Bk, continued line (*n* = 8); solvent, dotted line (*n* = 6). #Significant difference between profiles at *P* < 0.004 (repeated measurement ANOVA); * significantly different from pre-infusion control at *P* < 0.05 or less (Duncan’s post hoc test)

Effects of intramedullary Bk or solvent infusions on MAP, renal haemodynamic and renal excretion parameters in normotensive S-D rats are presented in Fig. 1. To assess if Bk effectively increased medullary perfusion (MBF), as intended, the profile of the induced change over time was compared to that in time-control experiments (solvent infusion) using multivariable repeated measurement ANOVA (Table 2). It is seen that a highly significant MBF increase (demonstrable also relative to the profile for the solvent) was observed. A major (+ 45%) Bk-induced increase in MBF was selective, i.e. not associated with any significant change in RBF, and was not associated with any significant change in MAP which remained stable over four hours. All parameters of renal excretion increased progressively and significantly. For V and U_Na_V (but not U_osm_V) the increase only tended to be greater with Bk compared to solvent infusion.

**Table 2:** Statistical comparison of the change in medullary perfusion (MBF) during 4 hof bradykinin versus solvent infusion into the renal medulla in the three experimental groups

Group	SS	MS	*F*	*P* <
S-D (7, 6)	9054	3018	5.3824	0.0040
S-D+NE (7, 8)	13751	4583	6.9015	0.0010
DOCA-salt (7, 7)	12967	4322	4.1004	0.0133

Comparison made using repeated measures multivariate ANOVA (post hoc Duncan’s test). S-D, normotensive Sprague-Dawley rats; S-D+NE, norepinephrine-induced hypertensive rats; DOCA-salt, hypertensive rats (n values for bradykinin, solvent). SS, sum of squares, MS, mean square, F value. The degree of freedom for interaction of time (t) × treatment (k) factor equals (n-t) (n-k), i.e. (4-1)(2-1) = 3 for each group

### Acutely hypertensive rats

**Fig. 2:**
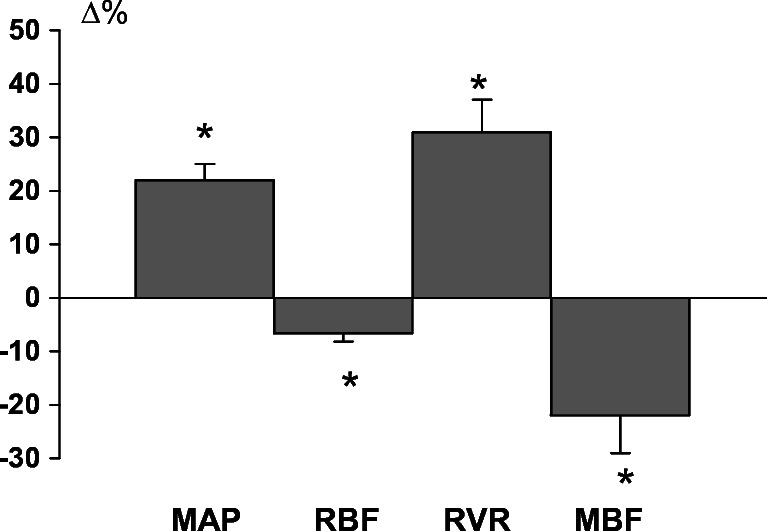
Effects of intravenous infusion of norepinephrine at 0.83 mg h^−1^ kg^-1^ BW on hemodynamic parameters in normotensive Sprague-Dawley rats (*n* = 6). MAP, mean arterial pressure; RBF, renal blood flow; RVR, renal vascular resistance; MBF, renal medullary perfusion. Mean Δ% ± SEM, *significant change from control at *P*< 0.05 or less (paired Student’s *t* test)

The study of S-D+NE rat group was preceded by examination in six rats of the feasibility of NE infusion as a tool for acute and possibly sustained elevation of MAP. Within 40-min infusion MAP stabilized at a level ~ 23% above the control, which was associated with a significant 7% decrease in RBF (Fig. 2). The calculated renal vascular resistance increased from 19.2 ± 1.3 to 25.1 ± 1.8 mmHg ml^−1^ min (+ 30%). MBF decreased more (− 23%) than did RBF.

**Fig. 3:**
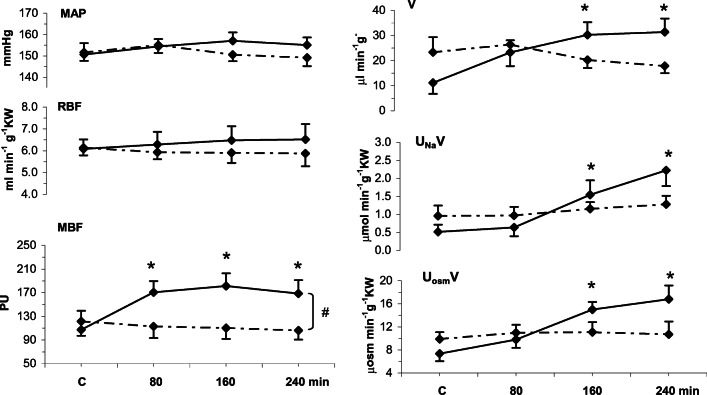
Responses of mean arterial pressure, renal haemodynamics and excretion parameters to 4-h renal intramedullary infusion of bradykinin (Bk) or solvent in rats with norepinephrine-induced blood pressure elevation MAP, mean arterial pressure; RBF, renal blood flow; MBF, renal medullary perfusion; excretion of water (V), sodium (U_Na_V) and total solutes (U_osm_V). Means ± SEM: averaged 30-min values measured in the control period (C) and at the end of 80, 160 and 240 min of Bk infusion. Bk, continued line (*n* = 7); solvent, dotted line (*n* = 8). #Significant difference between profiles at *P* < 0.001 (repeated measurement ANOVA); *significantly different from pre-infusion control at *P* < 0.05 or less (Duncan’s post hoc test)

In the main study of rats with NE-induced elevation of MAP (Fig. 3) intramedullary Bk increased MBF by 65% while with solvent infusion no significant increase was seen; the difference between the two MBF profiles was highly significant (Table 2). However, with both Bk and the solvent, MAP remained stable throughout the experiment. In Bk- but not in solvent-infused rats, V, U_Na_V and UosmV showed a progressive significant increase; the excretion profiles were not parallel to that of MBF.

### DOCA-salt rats

**Fig. 4:**
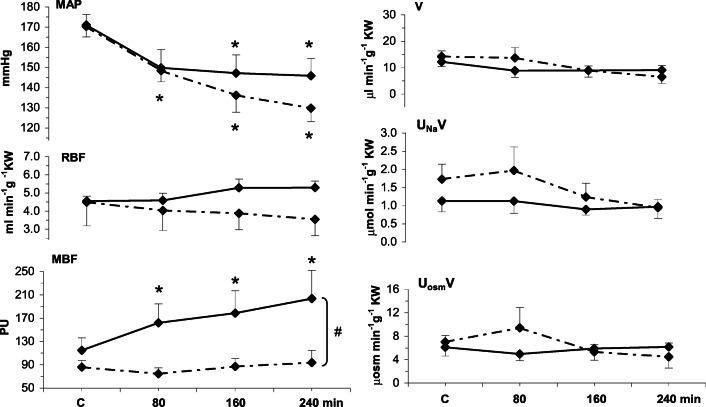
Responses of mean arterial pressure, renal haemodynamics and excretion parameters to 4-h renal intramedullary infusion of bradykinin (Bk) or solvent in rats with DOCA-salt hypertension model MAP, mean arterial pressure; RBF, renal blood flow; MBF, renal medullary perfusion; excretion of water (V), sodium (UNaV) and total solutes (UosmV). Means ± SEM: averaged 30-min values measured in the control period (C) and at the end of 80, 160 and 240 min of Bk infusion. Bk, continued line (*n* = 7); solvent, dotted line (*n* = 7). #Significant difference between profiles at *P* < 0.01 (repeated measurement ANOVA); *significantly different from pre-infusion control at *P* < 0.02 or less (Duncan’s post hoc test)

In rats with DOCA-salt hypertension, as in the two former groups, intramedullary Bk significantly increased MBF (+ 70%) while with the solvent the change was not significant (Fig. 4, Table 2). Unlike with normotensive and S-D+NE rats, MAP did decrease during experiment but the change tended to be more pronounced with the solvent than with Bk infusion. RBF and the renal excretion parameters did not significantly change with either infusion.

### Tissue studies

Effects of Bk and solvent infusion on total solute (osmolality) and Na^+^ concentration in the inner medullary tissue are shown in Table 3. It is seen that in each of the three rat groups both the mean osmolality and Na^+^ values appeared to be lower in the tissue of rats infused Bk, as compared with the solvent, but the Bk vs. solvent difference proved significant only for the Na^+^ value in normotensive S-D rats. A significant difference in tissue Na^+^ (but not osmolality) was also seen when the data for the three groups were pooled (*n* = 22): 124 ± 8 mmol kg^−1^ for Bk and 144 ± 6 mmol kg^−1^ for the solvent (*p* < 0.04), indicating a modest wash-out of tissue sodium during increased medullary perfusion.

**Table 3:** A comparison of tissue osmolality and sodium concentration [Na+] in the inner medulla, measured at the end of Bk and solvent infusion studies in the three experimental groups

	Medullary osmolality		Medullary [Na^+^]	
	(mosm kg^−1^ wet medullary tissue)		(mmol kg^−1^ wet medullary tissue)	
	Bk	Solvent	Bk	Solvent
S-D	614 ± 129 (8)	656 ± 42 (6)	123 ± 12* (8)	166 ± 7 (6)
S-D+NE	497 ± 49 (7)	551 ± 68 (8)	109 ± 8 (7)	123 ± 9 (8)
DOCA-salt	598 ± 71 (9)	673 ± 88 (8)	137 ± 21 (8)	149 ± 6 (6)

S-D, normotensive Sprague-Dawley rats; S-D+NE, norepinephrine-induced hypertension rats; DOCA-salt, hypertensive rats; Bk, bradykinin. Values are means ± SEM; n values are given in brackets

*Significantly lower than in the corresponding solvent infusion study. P < 0.05, unpaired Student’s t test)

## Discussion

While in the past three decades the main effort in evaluation of the role of MBF in control of BP focused on animal and human hypertension, the rate of medullary perfusion was also proposed to be involved in the regulation of pressure fluctuations under physiological conditions [13, 22].

The first important finding in this study was that in anaesthetised normotensive rats a 4-h increase in medullary blood flow did not induce any decrease in arterial blood pressure. This was so even though medullary hyperperfusion caused some modest wash-out of inner medullary sodium and renal sodium excretion at least tended to increase. No evidence for a fall in BP despite an almost 50% increase in MBF maintained during four hours indicates that within this time interval medullary hyperperfusion has no role in control of BP in normotensive animals. This extends our earlier observation on the absence of such hypotensive action of medullary hyperperfusion in three experimental models of rat hypertension [10] to include also physiological blood pressure control. Thus, the finding is not compatible with the long postulated role of medullary circulation changes to correct temporary (hour-to-hour) BP elevation in normotensive animals, e.g. in response to a fluid load [13, 22, 23]. In other words, our present findings put to doubt the hypothesis that in healthy animals an acute elevation of arterial pressure can be corrected within hours via an increase in—allegedly poorly autoregulated—MBF [13, 14, 22, 25]. Such scenario could not be excluded a priori because increasing perfusion of the medulla results in elevation of local interstitial hydrostatic pressure, which is very likely to alter the complex status of local endocrine, paracrine and neural influences (e.g. of angiotensin II, endothelin, prostanoids, nitric oxide and reactive oxygen species) [19]. Such changes could within a few hours induce also systemic effects, e.g. vasodilation and a decrease in blood pressure.

The findings do not bear directly on the postulate that medullary hyperperfusion, when maintained over days, lowers blood pressure as a result of natriuresis and extracellular fluid volume contraction. Clearly, longer lasting exposure to medullary hyperperfusion would be needed to examine whether an MBF change is or is not responsible for correction of blood pressure increase mediated by the pressure-natriuresis mechanism, e.g. in response to temporary or prolonged excessive salt intake.

In the next step of this work, as baseline situation for the study of the effects of medullary hyperperfusion we have chosen a moderate elevation of arterial pressure secondary to acute infusion of NE; such a model might be thought to resemble a border-line hypertension of short duration. We hoped to obtain circumstances in which the dominating clearly defined alteration, i.e. BP elevation would be countered, or not, by increased perfusion of the medulla. Within a 4-h experiment, the known multiple long-term consequences of NE excess (e.g. sodium retention or stimulation of renin release) might not yet have come into play. Indeed, during experiment, there was no sodium or water retention: in fact, the excretion increased, most probably due to the dominating effect of pressure natriuresis (Fig. 3). Not likely within that short time period was also any major stimulation of renin release and activation of the renin-angiotensin system (RAS), especially an elevation of plasma angiotensin II (Ang II). If present, it would probably be associated with an increase in RVR greater than the observed 30% elevation. In our earlier study, we demonstrated that with the doses of the two vasodepressors matched to induce the same acute BP elevation, RVR increased 45% with NE and 130% with AngII, indicating specific renal vasoconstrictor action of the latter [12]. Overall, our S-D+NE group should be described as one with “temporary or transient pressure elevation” rather than established experimental hypertension.

We found that under conditions of such moderate blood pressure elevation a substantial (65%) increase in MBF did not cause any decrease in arterial pressure, the result similar as observed with normal baseline BP level. Evidently, increased renal medullary perfusion was not a factor likely to cause a decrease in arterial pressure, similarly at normal and modestly elevated baseline pressure level. This finding is comparable with that of our previous study where we examined the response of arterial pressure to experimental renal medullary hyperperfusion in salt-loading hypertension, AngII-induced hypertension and in spontaneously hypertensive rats (SHR), a genetic model with fairly complex pathogenetic background [10]. It will be noticed that in the SD-NE rats (not in the two other models), the parameters of renal excretion increased during Bk-induced medullary hyperperfusion (Fig. 3), indicating some effect postulated by the renocentric theory. However, even though increasing, the excretion rates were lower than the total fluid infusion rate during the experiment so that the rats were retaining and not losing body fluid (positive balance). In conclusion, such minor and short-lasting natriuresis was wholly unlikely to have caused any significant ECF volume change which might affect blood pressure.

In the last step of the present work, we examined the response to renal medullary hyperperfusion in rat DOCA-salt hypertension, a model not explored in this respect in our earlier studies. The model consists primarily of sodium retention (related to increased sodium intake and inhibited tubular sodium reabsorption due to increased mineralocorticoid activity) and inhibition of renin release. However, the hypertension is also of neurogenic origin, apparently dependent on activation of the brain renin-angiotensin system (RAS) [4, 29] . Interestingly, DOCA-salt mice were reported to display diminished pressure-natriuresis, possibly dependent on abnormal response of MBF [20]. The DOCA-salt model can be thought to mimic, at least to some extent, the low-renin hypertension in humans.

In DOCA-salt hypertensive rats, a mean Bk-induced selective increase in MBF was substantial (+ 70%) and within first 80 min was associated with a distinct decrease in MAP. However, the fall was quite similar as that seen with solvent infusion where the same MAP decrease also occurred. Hence, no pathogenetic role can be ascribed to this initial BP decrease. Later during experiment in Bk-infused rats, MAP remained stable, and in the solvent counterparts, it decreased further. Unexpectedly, the progressing MBF increase in the former appeared to oppose the decrease in MAP observed in time-control experiments. On the whole, there was not the least evidence on MBF-mediated decrease in arterial pressure.

An obvious limitation of this study is that experiments were conducted under anaesthesia. Very likely, this was responsible for the decrease in BP observed during DOCA-salt group experiments. The reason might be a progressing inhibition by the anaesthetic of the diverse pro-hypertensive mechanisms activated during prolonged sodium retention and hyperactivity of the sympathetic nervous system; such activation is an established feature of the DOCA-salt model. Notably, no such pressure decrease was observed here in normotensive rats or in the group with recent acute BP elevation (S-D+NE group), i.e. when the mentioned mechanisms have not yet been activated. However, interpretation of the different response pattern is difficult. In normotensive and NE-infused rats, the baseline sympathetic tone must have been elevated as a result of anaesthesia and extensive experimental surgery (including acute right-side nephrectomy) and in NE-infused rats subjected to the same procedures, the actual status of the brain and peripheral sympathetic nervous system would be difficult to predict and would require another focused study.

In that and the present study of rat hypertension models, the decrease in BP can by no means be ascribed to the medullary hyperperfusion since a similar or even greater reduction of BP was observed with intramedullary solvent and no change in MBF. It will also be noticed that in our earlier studies comparable negative results (increased MBF, wash-out of medullary Na^+^ but no decrease in BP) were obtained in chronic (no anaesthesia) exposure of conscious rats to medullary vasodilator action of Bk [10].

In conclusion, the study provides new data indicating that a change in renal medullary perfusion is not a critical factor enabling correction of blood pressure fluctuations in normotensive animals or animals with modestly elevated blood pressure. Nor is it important in the pathogenesis of rat hypertension dependent on sodium retention associated with activation of the brain RAS, a model resembling in some respects the low-renin hypertension in humans. Together with earlier data, the rate of medullary perfusion is shown to have no role in control of arterial pressure in experiments with normotensive and hypertensive rats, at least when examined during the first 4-h period of MBF elevation.
